# Exploring the barriers and facilitators to use of point of care tests in family medicine clinics in the United States

**DOI:** 10.1186/s12875-016-0549-1

**Published:** 2016-11-03

**Authors:** Victoria Hardy, Matthew Thompson, William Alto, Gina A. Keppel, Jaime Hornecker, Adriana Linares, Beth Robitaille, Laura-Mae Baldwin

**Affiliations:** 1 University of Washington Department of Family Medicine, Seattle, WA 98195 USA; 2 Swedish Cherry Hill, Family Medicine Residency, Seattle, WA 98122 USA; 3 University of Wyoming Family Medicine Residency, Casper, WY 82601 USA; 4 Family Medicine of Southwest Washington, Vancouver, WA 98668 USA

**Keywords:** Point-of-care tests, Near patient testing, Family medicine, Infections

## Abstract

**Background:**

Point-of-care tests (POCTs) are increasingly used in family medicine clinics in the United States. While the diagnostics industry predicts significant growth in the number and scope of POCTs deployed, little is known about clinic-level attitudes towards implementation of these tests. We aimed to explore attitudes of primary care providers, laboratory and clinic administrative/support staff to identify barriers and facilitators to use of POCTs in family medicine.

**Methods:**

Seven focus groups and four semi-structured interviews were conducted with a total of 52 clinic staff from three family medicine clinics in two US states. Qualitative data from this exploratory study was analyzed using the constant comparison method.

**Results:**

Five themes were identified which included the impact of POCTs on clinical decision-making; perceived inaccuracy of POCTs; impact of POCTs on staff and workflow; perceived patient experience and patient-provider relationship, and issues related to cost, regulation and quality control. Overall, there were mixed attitudes towards use of POCTs. Participants believed the added data provided by POCT may facilitate prompt clinical management, diagnostic certainty and patient-provider communication.

Perceived barriers included inaccuracy of POCT, shortage of clinic staff to support more testing, and uncertainty about their cost-effectiveness.

**Conclusions:**

The potential benefits of using POCTs in family medicine clinics are countered by several barriers. Clinical utility of many POCTs will depend on the extent to which these barriers are addressed. Engagement between clinical researchers, industry, health insurers and the primary care community is important to ensure that POCTs align with clinic and patient needs.

**Electronic supplementary material:**

The online version of this article (doi:10.1186/s12875-016-0549-1) contains supplementary material, which is available to authorized users.

## Background

Point-of-care tests (POCTs) are diagnostic tests conducted by non-laboratory trained staff (e.g. physicians and their assistants) near to the patient [1, 2], in many primary care settings worldwide. Most family medicine clinics in the US have a small on-site laboratory, which provides facilities to undertake diagnostic tests (including POCTs) categorized by the US Food and Drug Administration (FDA) as being of low complexity to implement and interpret (referred to as CLIA-waivered tests) [3]. These laboratories conduct tests such as Rapid Group A Streptococcus antigen tests or dipstick urine tests, as well as obtain specimen samples for sending to external laboratories for more complex testing.

Family physicians in the US routinely use support staff called Medical Assistants (MAs) to assist with many of their daily administrative and clinical tasks, which include greeting patients, updating medical records, measuring vital signs, and conducting CLIA-waivered tests. Depending on the regulatory and accreditation standards governing POCTs [4], they are undertaken either in on-site clinic laboratories immediately after a patient visit (requiring the patient to check back in with the physician), or in the physician’s office by the physician during the patient visit or by the MA ahead of medical review by the physician [5]. This contrasts with POCTs undertaken in countries such as the United Kingdom that are mostly done in the physician’s office by the physician [6].

POCT results can typically be obtained within a short period of time (e.g., 10-min for hemoglobin A1c) [7], allowing clinical management decisions and treatment changes to be made at the time of the patient visit [5]. In the US family medicine setting, this could circumvent fragmentation of patient care and delayed communication of test results to patients, currently caused by waiting for samples to be processed and results returned from internal and external laboratories [2, 5, 8]. Furthermore it could reduce the need and costs of additional office visits [9]. Finally, by using POCTs, clinics could absorb some of the testing currently performed by off-site laboratories, potentially reducing health care costs [10, 11].

The range of POCTs available has grown rapidly in recent years [11], yet relatively few are in widespread use in primary care settings in the US. A systematic review of qualitative studies conducted in Europe and Australia highlighted clinician concerns about inaccuracy, cost and quality control as factors in deciding whether to adopt POCTs [12]. Unique differences in health care models between Europe and the US in terms of insurance systems, clinic reimbursement, diagnostic test regulations, clinic organization and staffing may lead to different constraints and demands for implementing POCTs. Currently there is no comparable data on factors that may be impacting adoption in the US. We therefore aimed to explore the attitudes of primary care providers, laboratory and clinic administrative and support staff towards POCTs, to identify the barriers and facilitators to use in family medicine clinics in the US. We deliberately chose to include a variety of clinic staff to provide a broader understanding of the issues associated with delivering POCTs in these settings.

## Methods

### Sampling and recruitment

This qualitative study used focus groups and semi-structured interviews to understand the barriers and facilitators towards use of POCTs. An inductive exploratory approach was employed due to fragmented knowledge of the phenomenon related to the setting [13]. We identified and included three primary care clinics in the WWAMI region Practice and Research Network (WPRN); a practice-based research network in the 5-state Washington, Wyoming, Alaska, Montana and Idaho (WWAMI) area comprising over 50 family medicine clinics. Research sites were selected based on our desire to include clinics with a range of characteristics such as location (i.e. urban, small town settings) and type (i.e. private health system-based clinics and publicly-funded health centers known as Federally Qualified Health Centers), and to include clinics serving populations with varied clinical needs and diverse experiences of POCTs. The included sites serve patients who have private and government-regulated health insurance and/or who are means tested, and thus pay a reduced fee to cover health service costs, including payment for POCTs. Participants included primary care providers (i.e. family medicine physicians and resident physicians in training, pharmacists, nurse practitioners); laboratory staff, and clinic administrative and support staff (i.e. clinic administrators, medical assistants, nurses). Clinic selection was coordinated by the WPRN Coordinating Center, which communicated study information (including eligibility criteria)–verbally and in writing–to clinic ‘research champions’ (i.e. clinicians acting as the primary point of engagement between WPRN clinics and the research team). The research champions conveyed study information, which included the time and location of scheduled focus groups/interviews (typically protected staff meeting time within clinics), to clinic staff through email/at staff meetings. Self-selection sampling was used whereby interested staff whom were eligible and available at the time of focus groups/interviews volunteered to participate.

### Data collection

A mixture of focus groups and one-to-one semi-structured interviews were conducted. Focus groups were the primary method of data collection, as they stimulate a broader range of responses than interviews. Interviews were used to provide richer insights into emerging themes, and when there were very small numbers of participants fulfilling specific job roles (e.g. laboratory technicians) [13].

Focus groups and interviews were held at participants’ workplace between January and July 2015. To encourage open and inclusive discussions [14], focus groups were conducted according to job role where possible. Interviews were conducted with two laboratory technicians and two clinic administrative support staff. Three focus groups included participants that were heterogeneous; one included a mixture of clinic administrative support staff and providers.

Three semi-structured topic guides were created by the study team and tailored for each job role (i.e. providers, clinical administration/support staff, and clinic laboratory staff). They included semi-structured open-ended questions (with prompts) relating to: participants’ general opinions of POCTs; POCTs currently used in clinical practice, and future use and attitudes towards POCTs (see Additional file 1). Topic guides assumed a semi-structured format to allow flexibility to focus conversations about specific topics and to follow new concepts as directed by participants, and as required during focus groups and interviews. Additional questions were added to the guides (during analysis) as interviews progressed. Topic guides were reviewed by clinic research champions prior to use to verify suitability of material, but were not pilot tested.

Interviews were conducted by one author (MT, a Family Physician with prior experience conducting qualitative interviews). The interviewer did not know participants at the time of data collection. Participants were informed the interviewer was a Family Physician during the opening remarks of each session, at which time an overview of the study was also given. Focus groups and interviews were audio-recorded and lasted between 45–60 min and 20–30 min, respectively.

All participants were given a brief survey at the close of interviews to collect information on job title, age, gender, year education or training for current profession/position was completed, and years worked at the current clinic, to enable us to describe the study sample.

### Data analysis

Focus groups and interviews were transcribed verbatim and cross-referenced with audio-recordings to verify accuracy and remove identifiers. Data analysis began after the first focus group/interview had been transcribed and progressed concurrently with data collection. Analysis began with two authors independently reading the transcripts to familiarize themselves with the data; one author simultaneously listened to the audio-recordings for better context. Themes were identified using the constant comparison method (CCM) of grounded theory. CCM was utilized as it offers a systematic approach to synthesizing data and deepening enquiries.

Sections of data were coded into categories using open coding; categories were tested for “fit” against data from subsequent interviews, modified, and grouped into descriptive themes [14]. Assignment of data to categories and categories to themes was undertaken by VH and contrasted with those identified by MT at regular intervals during analysis. Categories and overarching themes were adjusted as data analysis progressed according to discussions between the authors. Data from focus groups were analyzed first and separate to semi-structured interviews, starting with the first participating clinic site. Due to overlapping themes the coding structure was subsequently combined.

To minimize potential bias, data analysis was led by a non-clinical researcher (VH) not present during data collection. Focus groups/interviews ceased when data no longer contributed new information to categories/themes [14]. All authors reviewed the final themes to check for consensus. The ‘One Sheet Of Paper’ (OSOP) technique was used following development of the coding structure to further explore and connect patterns within themes to facilitate write-up of the results. During OSOP the various issues elicited by the coded excerpts from transcripts within each category are manually summarized on one piece of paper (along with the participants’ ID number) [15]. NVivo 10©, was used to organize the data ﻿during analysis.

## Results

### Participants

We conducted seven focus group discussions and four semi-structured interviews, which included a total of 52 participants, of whom 45 were primary care providers, five filled clinic administrative or support staff roles (including two with a joint clinical and laboratory support role) and two filled laboratory roles (Table 1). All participants attending focus groups/interviews agreed to participate; none declined involvement.

**Table 1:** Characteristics of participants

Characteristics	*N* = 52 (%)
Clinic role	
Providers	
Physician	20 (38.5)
Resident Physician	20 (38.5)
Pharmacists	2 (3.8)
Nurses	3 (5.8)
Clinical administrative and support staff	5 (9.6)
Laboratory staff	2 (3.8)
Gender	
Male	25 (48 %)
Mean age, years	42 (26–74)
Mean years at clinic	6.7 (0.50–35)
Median year completed education for current role	2004

### Barriers and facilitators to use of POCTs

Five major themes emerged from the data regarding barriers and facilitators to use of POCTs, namely:

- Impact of POCTs on clinical decision-making
- Concerns about perceived inaccuracy of POCTs
- Impact of POCTs on perceived patient experience and patient-provider relationship
- Impact of POCTs on staff and clinic workflow
- Influence of regulation, quality control and cost [on use of POCTs]

We present each of the above themes in detail (summarized in Table 2). To preserve participant confidentiality abbreviated quotes are protected using interview identification numbers, with letters to delineate type of respondent (e.g. Physicians, Resident physicians, Administrative/support staff, Laboratory) followed by clinic site number (e.g. S1, S2, S3). Full quotations supporting the themes are sequentially numbered within the narrative and are presented in more detail (see Additional file 2).

**Table 2:** Summary of the facilitators and barriers to use of point of care tests (POCT) in family medicine clinics

Themes	Facilitators	Barriers
1. Impact on clinical decision-making	- Faster decision-making - Earlier triaging of possible serious illness - Improved confidence in treatment decisions (e.g. antibiotics)	- Immediate results not helpful in some situations (e.g., monitoring of chronic conditions) - Over-reliance undermining physician clinical skills - Increase in unnecessary testing
2. Accuracy concerns	- Improved ‘rule out’ value when used with clinical features	- Less accurate than laboratory tests - Positive test results often misleading
3. Impact of POCT on staff and clinic workflow	- Reduced clinic difficulties with patient follow-up for laboratory tests between office visits - More POCT may alleviate pressure on under-staffed laboratories	- Concerns increased testing volume may extend patient visits/overwhelm providers - Insufficient healthcare personnel within clinics to manage additional testing - Risk of error in reporting results for tests without EMR interface
4. Impact on perceived patient experience and patient-physician relationship	- Improved patient-provider communication - Patient awareness of work involved in making a diagnosis, making providers feel more valued - Improved patient understanding and acceptance of provider treatment decisions (e.g., antibiotics) - Perceived greater acceptability of fingerstick blood testing by patients and clinic	
5. Influence of cost, regulation and quality control		- Perceived expense compared to laboratory tests - Uncertainty about reimbursement rates from insurers and loss of clinic revenue - Lack of laboratory trust in giving clinics responsibility for quality control processes - Lack of clinic autonomy to adopt new tests

#### Impact of POCTs on clinical decision-making

Faster decision-making was frequently cited as an advantage to using POCTs amongst all participants, particularly providers. Having the test result available immediately enabled providers to make treatment decisions or changes in clinical management, avoiding the need to wait until test results were returned from an external laboratory. This characteristic was seen as potentially most valuable for acute triage for serious issues (e.g. possible sepsis) as providers could make quicker decisions about whether a higher level of care was needed, or, it would give them additional data to have “*an idea of which way to go*” (R, S1) to get the patient on the right track if they were concerned a patient was critically ill (**Q1**). Being able to make a determination quickly was perceived as enhancing patient safety and being more efficient (**Q2**). Participants added that the value of POCTs in guiding decision-making was limited if tests yielded intermediate results (i.e. neither positive nor negative), which they had experienced previously with point-of-care troponin and d-dimer tests. However, for monitoring other conditions, such as chronic illnesses, where time to consider treatment options can be helpful (**Q3**), it “*might not be advantageous to know that result with the person sitting across from you*” (P, S1).

Several providers expressed confidence (which some clinic support staff also recounted) in the use of clinical signs and symptoms to guide treatment decisions on whether to treat or not treat (both types of decisions were considered important) for commonly presenting illnesses such as respiratory infections, and identified scenarios where POCT may provide limited added value; for example, some felt they should be able to diagnose streptococcal tonsillitis based on clinical findings alone, and that a rapid antigen test for group A streptococcus would not alter their decision-making, regardless of the result “*because you should be able to tell, and you’re going to treat it the same anyway*” (A, S1). For these conditions, participants cautioned against over-reliance on testing which they believed could undermine providers’ clinical skills and usefulness (**Q4**). Nevertheless, POCTs were recognized as beneficial for situations where there was (continuing) uncertainty, for example discerning bacterial from viral infections in atypical presentations, or where there was perceived pressure from patients to prescribe (e.g. antibiotics) (**Q5**). For these types of scenarios POCTs were identified as useful for supporting prescribing decisions (**Q6**).

#### Concerns about perceived inaccuracy of POCTs

Another frequently occurring theme amongst participants across clinic roles, was the perception that POCTs were less accurate than routine laboratory tests. There was an assumption that even though they allowed providers to make on-the-spot clinical decisions, POCTs were not as accurate. These perceptions were partly the result of past experiences of inconsistencies between point-of-care and laboratory results (**Q7**; **Q8**). Concerns about inaccuracy were predominantly focused on getting false positive results from POCTs, which had occasionally misled participants in the past, resulting, for example, in a medication incorrectly being given. There was overall more inclination towards trusting negative results as rule-out tests, rather than positive results to rule in a condition (**Q9**). Consequently, participants reported that “*almost all of the ones (POCTs) we do are followed up by a confirmatory (laboratory) test*” (R, S3) especially if the test is positive, in order to be confident of the result (**Q10**). Providers believed POCTs could be used to rule in a condition provided they are used when clinically indicated, and in conjunction with clinical guidelines or findings, to increase the test’s specificity (**Q11**). Availability of false positive and false negative results with guidance on when the test should be used and how to interpret results, presented in summary format and pertaining to individual POCTs, would enable clinic staff to better understand the strengths and limitations of tests, which would offset imperfect diagnostic accuracy of POCTs.

#### Impact of POCTs on staff and clinic workflow

A perceived benefit of POCTs, which was linked to immediate knowledge of the test result, was the potential for streamlining patient care. Many participants expressed difficulties contacting patients to communicate results between visits, or arranging follow-up laboratory testing (**Q12**). Following-up hard-to-reach populations (e.g. individuals who are homeless, minority populations) was highlighted as a major problem due to lack of fixed address, incorrect or no telephone numbers, and language barriers (**Q13**). This also applied to patients living in more remote geographic settings or with busy work schedules, who were described as often unable to take further time off work to travel lengthy distances for additional office visits, or to be reached by follow-up telephone calls.

There was, however, apprehension about the impact increased use of POCTs might have on providers’ and clinic administrative/support staff workload during patient visits. For patients presenting with multiple problems, providers felt they may be placed under pressure to test and manage each complaint there and then, extending patient visits and overwhelming staff (**Q14**). Similarly, lack of health care personnel to run POCTs was identified by clinic administrative/support staff as impeding workflow, as POCTs were “*another thing that has to be done by someone*” (A, S2). Some clinics did not have high levels of staffing for their clinic laboratories, or sometimes no dedicated laboratory technician, meaning medical assistants (MAs) performed some laboratory duties within two of the practices, in addition to their primary role. There were mixed opinions about the feasibility of having MAs (or other support staff) undertake more testing. Providers were sensitive towards additional workload and time pressures more POCTs might impose on support staff as it would potentially “*pull her [the MA] out of the rotation for 10 minutes, when they could be rooming the next patient*” (P, S2). This seemed to be less of a concern for support staff themselves, who were motivated by their role in using POCTs to be “*that one step ahead*” (A, S3) for their provider (**Q15**). From the laboratory staff perspective the fact that POCTs could be performed by other staff, was considered an advantage, as it could ease their workload (**Q16**; **Q17**).

Without an interface automating integration of results into electronic medical records (EMR), there were concerns expanding POCT use within clinics may compound pressures on clinic administrative/support staff, and that manual data entry had already led to results being erroneously entered and reported in the past (**Q18**; **Q19**). For one site, lack of health care personnel and absence of seamless data entry features were primary reasons for not adopting certain POCTs (**Q20**).

#### Impact of POCTs on perceived patient experience and patient-provider relationship

Providers felt POCTs helped improve communication with patients and were an opportunity for health education (**Q21**). Providers believed patients did not always understand test results when they received them in a letter, so being able to have that discussion with them in real time was viewed as giving patients the opportunity to seek clarification, which might help improve understanding of their clinical management or provide reassurance (**Q22**). Additionally, one participant stated that giving patients results directly “*from the horse’s mouth*” (meaning the provider) might make patients appreciate that “*we really are trying to help ‘em out*” (P, S3), making providers feel their efforts to alleviate patient concerns/symptoms is better understood. Moreover, providers believed an immediate test result might improve patient acceptance of a provider’s treatment decisions. Providers described pressure for antibiotics and particular situations where providing patients with a tangible result could substantiate providers’ decision-making, without negatively affecting patient satisfaction (**Q23**).

Participants, irrespective of job role, perceived certain characteristics of POCTs as more acceptable to patients. Notably, the ease of blood sampling with finger stick POCTs was viewed as more convenient to participants and believed to improve patient compliance. Participants reflected on experiences where patients had explicitly favored finger stick testing and were felt to cause less anxiety than a venous draw due to the needle being hidden from view. Consequently, participants identified broader utility of this type of test (e.g. drug users with collapsed veins), which would facilitate clinical practice (**Q24; Q25**).

#### Influence of cost, regulation and quality control on use of POCTs

A major barrier to POCTs was perceived cost to the clinic. For certain POCTs (e.g. HbA1c) the cost per test was thought to be greater than send-out laboratory tests, though most providers did not know exact comparative costs, and acknowledged that costs varied depending on the test (**Q26**). Perceived higher costs was thought to be due to the up-front cost of purchasing analyzers and test strips/reagents and the labor involved in their set-up and daily running (e.g. staff training, quality checks, data entry procedures). In addition, lack of standardized reimbursement rates across insurers left participants unclear about the overall cost-effectiveness at the clinic-level of POCT (**Q27**).

Laboratory staff had reservations about quality control and calibration procedures for POCTs, such as who does them (in-house, or externally) and how frequently (**Q28**). There was skepticism by the laboratory technicians regarding how realistic it would be for quality control procedures to be conducted in-house by clinic staff, and whether laboratories would be comfortable relinquishing control of centralized testing. Usability of POCTs and consistency in how they are conducted by various clinic staff was felt to contribute to quality control concerns (**Q29**).

In terms of implementing POCTs, two clinics noted a lack of autonomy regarding decisions to adopt new tests, one of which described a perceived resistance by the clinic’s overarching healthcare system and its central laboratories to clinic-based testing, describing lengthy previous attempts (between 6 months and 2 years) getting new POCTs approved for clinic use (**Q30**).

## Discussion

### Main findings

To the authors’ knowledge, this is the first study to explore attitudes to use of POCTs in family medicine settings in the US. Exploring the perspectives of a variety of clinic staff, provided insight into the clinical, operational and financial barriers and facilitators to POCT. Overall providers, clinic administrative/support and laboratory staff held mixed and overlapping opinions about use of POCTs in family medicine clinics.

There was a general belief that additional data yielded by POCTs facilitated decision-making in scenarios where there were concerns that patients may be acutely unwell, as well as when there was diagnostic uncertainty or perceived pressure from patients for certain treatment(s) (e.g. antibiotics). Other facilitators included reduced need to telephone and schedule follow-up visits with patients following visits, and real-time communication with patients about their illness, fostering acceptance and reassurance in the providers’ treatment decisions. There were concerns about perceptions of inaccuracy of POCTs amongst some clinic staff, stemming from inconsistences in test results between POCT and laboratory tests. Participants seemed to place greater trust in negative than positive POCT results, and in many cases used additional laboratory testing to confirm ‘positive’ results. Providers were hesitant about the practicalities of introducing more POCT; highlighting insufficient staff to accommodate more testing, and lack of automated integration of POCT results into the electronic medical records (EMR) as barriers to uptake. Conversely clinic support and laboratory staff believed POCT enhanced patient workflow during office visits, and circumvented laboratory staffing constraints. Other barriers included worry about the impact more POCT might have on already time-constrained patient visits, and uncertainty about the overall cost-effectiveness of POCT.

### Comparison with existing literature

Existing research related to clinician attitudes towards POCTs has almost exclusively been conducted outside of the US. Nonetheless, our findings share many similarities with international studies. Notably, in a systematic review of qualitative studies on point-of-care blood testing conducted in European and Australian primary care settings, immediate results were identified as potentially reducing the need and inconvenience of time-consuming follow-up communication (e.g. letters and telephone calls) and patient visits for the same illness episode, as well as enabling real-time clinical management and communication of that plan to patients; improving patient satisfaction [12]. Similarly clinic staff in our study reinforced concerns expressed in other international studies regarding the diagnostic accuracy of POCTs [8, 9] and lack of confidence in test results (primarily relating to the sensitivity of tests) [12, 16]; and the resultant potential impact on length of patient visits , staff workload [9] and clinic workflow [1, 16]. Furthermore, fingerstick blood tests have been reported as acceptable to parents of acutely unwell children [17] and associated with high patient satisfaction [18], in line with participants’ perceptions in this study.

### Strengths and limitations

We used robust qualitative techniques and a sample of respondents from several different types of clinics and settings to gain an in-depth understanding of a range of issues related to implementation of POCTs in US family medicine clinics. To encourage neutrality and ensure views presented reflect participants, as opposed to the interests and biases of the interviewer, a researcher unfamiliar with POCTs and thus without preconceived expectations led data interpretation and analysis. While we had a greater number of clinicians and fewer administrative/support and laboratory staff in our study, themes emerging from focus groups with providers overlapped considerably with the semi-structured interviews with the smaller numbers of clinic administrative/support and laboratory staff, and so we do not consider the analysis of our data to be limited by differences in numbers of types of participants and sampling procedures. Whilst six of our focus groups included participants with shared characteristics (i.e. job role), due to our sampling strategy we were unable to achieve homogeneity at all sessions. Given the hierarchical structure of medical practices, this heterogeneity could have made participants hesitant to speak freely; in this instance we believe any potential negative impact was offset by even representation of providers and clinic support staff. We explored attitudes towards POCT in general, rather than focusing on a single POCT or intentionally exploring POCTs for every type of condition (which was beyond the scope of this study); the rationale being to understand the salient features of POCTs that are inhibitory or facilitative. Where specific POCTs were highlighted in relation to key issues identified, they have been presented. Different clinics and participants had varying experience with POCTs in their sites, reflecting the reality of POCT use in primary care, which may have contributed to differing positive and negative experiences with individual tests. Interviews were conducted by a clinical researcher with expertise researching and using POCTs, so in order to minimize bias caused by projection of personal agendas during data synthesis, the coding structure was developed by a non-clinical researcher with minimal research experience related to POCTs; all authors reviewed the themes (with coded extracts) to validate interpretation. Finally, we included five sites, and whilst sites were spread across two US states, we acknowledge our findings may not be representative of views towards POCT in primary care elsewhere in the US.

### Implications for clinicians, researchers and policy makers

While the point-of-care diagnostics market is substantial and growing, there has been surprisingly little research into end-user perceptions of the barriers and facilitators to POCT implementation. This research gap may be preventing more widespread uptake of POCTs in family medicine settings, as available POCTs may not be fully aligned with clinic users’ requirements. To harness their potential, industry should engage with clinicians during POCT development to align innovation with user requirements. There is mixed evidence regarding the cost-effectiveness of POCTs, with variation influenced by the POCT (and the related condition) and the setting [19]. Cost-effectiveness needs to be further considered, particularly in the context of poor user confidence in diagnostic accuracy of POCTs, prompting repeat confirmatory testing, as reported in this study. Furthermore, with an estimated 20 % of the U.S population living in rural areas with poor access (over 20 miles) to a primary care provider [20], there is a need to ensure patient, as well as clinic workflow, is optimized. Finally, diagnostic accuracy data should be made available to clinics, with guidance on how test results should be interpreted.

## Conclusions

There are numerous potential benefits to family medicine clinics from using POCTs. However, there are several important performance, organizational and financial challenges to optimizing delivery of POCTs, which need to be addressed for clinics to adopt more POCT, and for widespread deployment of these technologies in US primary care settings.

## Additional files

Additional file 1: Topic guide. Questions used in focus groups/semi-structured interviews. (DOCX 17 kb)
Additional file 2: Quotations supporting themes. Participant quotes illustrating themes. (DOCX 25 kb)

## Abbreviations

CLIA-waivered: Clinical laboratory improvement amendment
EMR: Electronic medical records
MA: Medical Assistant
OSOP: One sheet of paper
POCT: Point-of-care tests
US: United States
WPRN: WWAMI practice and research network
WWAMI: Washington, Wyoming, Alaska, Montana, Idaho
