# Supplementary material for: Exploring the barriers and facilitators to use of point of care tests in family medicine clinics in the United States
Source: BMC Fam Pract. 2016 Nov 3;17:149. doi: 10.1186/s12875-016-0549-1 (PMC5093922; doi:10.1186/s12875-016-0549-1)
Supplement: Additional file 1: — Topic guide. Questions used in focus groups/semi-structured interviews. (DOCX 17 kb) [file 12875_2016_549_MOESM1_ESM.docx]

**Supplementary File 1: Focus group/interview topic guide**

**General opinion of Point of Care Testing**

1. How do you feel about use of POCTs in primary care?
2. What are the perceived concerns and benefits [if any], you/other clinic staff have about POCTs

Prompts:

-Accuracy of test

-Workflow

-Patient satisfaction etc.

**Existing Point of Care Testing**

1. What point-of-care tests [if any] are available/do you use in this clinic?

Prompts:

-For which condition(s) are POCTs used?

-For what purpose is the POCT used (e.g. monitoring, diagnosis, referral)?

-How frequently is the POCT used for this condition?

-How are patients notified of their results?

1. What are the barriers and facilitators you experience conducting these POCTs?

Prompts:

-Is there anything that the clinic/laboratory does or could do to make the use of available/additional POCTs easier or more convenient?

**Additional Point of Care Testing**

1. Would you like to see more POCTs used in your clinic? Why/why not?

Prompts:

-Are there any POCTs that staff have asked for?

1. What do you think are the barriers/facilitators to adopting additional POCTs in your clinic?

Prompts:

-Are there specific types of POCTs you think would be particularly difficult/easy to adopt here? Why?

-What are the staffing/physical space/other implications of introducing (new or more) POCTs in your clinic?

-What, if anything, do you think this clinic could do to overcome barriers to adopting POCTs?
