# Supplementary material for: Exploring the barriers and facilitators to use of point of care tests in family medicine clinics in the United States
Source: BMC Fam Pract. 2016 Nov 3;17:149. doi: 10.1186/s12875-016-0549-1 (PMC5093922; doi:10.1186/s12875-016-0549-1)
Supplement: Additional file 2: — Quotations supporting themes. Participant quotes illustrating themes. (DOCX 25 kb) [file 12875_2016_549_MOESM2_ESM.docx]

**Supplementary file 2: Quotations supporting themes**

| **Theme 1: Impact on clinical decision-making** | |
| --- | --- |
| **Q1** | *“If a patient hasn’t been seen here for a long time, an A1C is invaluable. I mean, that will help confirm the need to start insulin.” (P, S2)* |
| **Q2** | *“you can have a situation where you get a critical value back in the middle of the night and you can’t get ahold of someone and… so I think it’s more efficient for the provider, it’s safer for the patient and I think it’s easier to handle more in that visit versus having to bring them back for a subsequent visit (P, S3)* |
| **Q3** | *“For acute issues, I think it's better.... for long term chronic care issues, like you said it's almost worse. cause for the acute care ones you want the answer right away, do I need to put this person on antibiotics, do I need to do something right now, versus long term care ones sometimes you want longer” (R, S3)* |
| **Q4** | *“I think we want to depend on them sometimes more than we should and it is a very slippery slope to get into. In the ERs I see a lot with the shotgun approach, you lose some of your clinical skills, or you doubt some of those clinical skills that should take actually precedence over some of that testing.” (R, S1)* |
| **Q5** | *“if you're kind of ... is this bacterial, is this viral, you know ... this person's sort of somewhat sick, but not that sick and you're trying to waffle ... antibiotics or no ..., I think you'd be probably more inclined if you had the rapid CRP back and it was more substantially elevated, you'd probably be more inclined to give that person antibiotics. So I can see it being a useful clinical tool in that way.” (P, S3)* |
| **Q6** | *“I think it helps because […] over the past 10 years how much has our antibiotic prescribing gone up? And so this kind of gives us an idea, ok we can tell the patient listen if it’s negative we’ll give it, […] or clinically yeah you know what… it does look like strep I will treat it” (R, S1)* |
| **Theme 2: Concerns about perceived inaccuracy of POCTs** | |
| **Q7** | *“My concern about the point of care A1C is that […] there is a discrepancy in the result […]. And it can be a major discrepancy, up to two points.” (P, S2)* |
| **Q8** | *“At times we've questioned accuracy in the coumadin clinic of our INRs, but I think we've worked through that for the most part... and part of that, too, is discrepancy, um, from our reference lab. So, we would do a quality check and those values would come back significantly different” (A, S1)* |
| **Q9** | “*If you get a negative, you’ll get a negative. If you get a positive and then that could be a false positive*” (A, S3). |
| **Q10** | *“If you do a hemoglobin, then if it's screened positive, then you're going to send the stuff to the lab as well. So a lot of times you end up doing two tests instead of one” (R, S3).* |
| **Q11** | *“I think also that’s helpful is like for instance with the rapid strep you know a lot of us won’t even use the rapid strep unless they meet like 4 certain criteria; like sore throat, no cough, fever, lymph nodes. You know like having those types of things along with, I mean that kind of helps...cause that increases its specificity I would say” (R, S1)*. |
| **Theme 3: Impact of POCTs on staff and clinic workflow** | |
| **Q12** | *“I would prefer not to have to waste time calling them afterwards, I… would rather see the person, get the test, discuss it with them right then and there… and then I’m finished with them. I don’t have to make my nurse call them and I don’t have to think about calling them and it doesn’t clog up my desktop. I just deal with it right then.” (P, S1)* |
| **Q13** | *“You get test results, and you try to phone them. You can’t phone them. You send them a letter, but you’re not certain that’s the right address, because they’re couch surfing, or they’ve moved on.” (P, S2)* |
| **Q14** | *“It could actually be a little bit of a hindrance sometimes, because you’re seeing them for one complaint and then they do the test and maybe it’s positive, maybe it’s negative but now you have to address it at that time. So now it’s actually extended, versus calling them back and having a separate visit.” (R, S3)* |
| **Q15** | *“We’re all here to help the doctors, make their job easier and have all the information they need […] so they start the conversation with, you know, how do we fix this problem, instead of what’s going on.” (A, S3)* |
| **Q16** | *“The point of care tests help the workflow because […] we have two techs and the other gal’s out…and so it’s like when I’m doing all that stuff and so when they [MAs] can do that, that really helps.” (L, S1)* |
| **Q17** | *“We only have one lab tech for this busy clinic and we’re about 200 visits a day.” (A, S3)* |
| **Q18** | *“When I’ve had mistakes, it’s always been because the MA had a million other things to do and typed it in, but typed it in wrong. Because her job is not to all day just sit there and input results, it’s you know, there are other things.” (R, S3)* |
| **Q19** | *“I think more than anything else, it uh, the results seem to get mixed up. You might get somebody else’s point of care.” (P, S2)* |
| **Q20** | *“One of the reasons we haven’t done point of care A1C’s for our relevant visits for diabetes is because there is no way our lab could kind of keep that up, so then it would kind of go on to the nursing to… maybe do the tests, and then not just do the tests, but then have to input the results in the EMR, because it wouldn’t be an automatic thing, and so that’s where we’ve kind of held off… is manpower.” (A, S1)* |
| **Theme 4: Impact of POCTs on perceived patient experience and patient-physician relationship** | |
| **Q21** | *“I think health education. I mean it's an opportunity right there. You know, people are here, you know, they're focused on their health, okay, and this is kind of an educational opportunity, you know, so I, I think it's good that way.” (A, S2)* |
| **Q22** | *“People get sent to me […], I think my blood sugar level is too high or these sugars are whacky and then you get an A1C and you’re like, no, your A1C is just fine.” (P, S3)* |
| **Q23** | *“It would be nice to have a viral panel where you could just tell someone, because they never believe you. You know, you have a virus antibiotics won’t help. If you could name the virus I think people would be so much more satisfied… And they would say, oh there’s a test that identified that” (P, S1)* |
| **Q24** | *“They would love a finger-prick before venipuncture procedure. […] I’ve had many patients request that actually […]they’re like oh no, I just want the finger check […] I don’t want any blood work, I’m afraid of needles (L, S3)* |
| **Q25** | *“There’s quite a few of them that we run and their veins are just shut, and there’s nowhere else we can get them. If there’s a possibility we could do point of care for those type that would be great, because […] some of them that come in, I mean we can’t draw them, because those veins are just shot (R, S3)* |
| **Theme 5: Influence of cost, regulation and quality control** | |
| **Q26** | *“My concern about the point of care A1C is that it is much more expensive than the draw sent to the [laboratory] […], uh, if it’s sent to the [laboratory] I think it’s like approximately 1/3 of the cost of a point of care here.” (P, S1)* |
| **Q27** | *“It depends on who’s paying… for Medicare, Medicaid, you typically lose money. Um, you may get reimbursed at a level that’s more than the cost of the…kit, but by the time you include all the other… costs that are associated with doing that test, and reporting that test, you’ve lost money.” (P, S2)* |
| **Q28** | *“When the nurses like do their glucometers and I know we check the QC [quality control] before, and I never addressed it, but I don’t know how often they check their QC.” (L, S1)* |
| **Q29** | *“Education of how to use [the equipment]… because not everyone is using it the same way, or not everyone is trained.” (L, S3)* |
| **Q30** | *“I’ve tried to get new tests approved at the… at the hospital, and it’s generally about… the process for me has been between 6 months to 2 years, […] so you just have to get buy-in from the lab people here, but then send it to a lab committee down there. Um, so it’s a pretty painful slow process.” (P, S3)* |
